# Meta-analysis on the association of *VEGFR1* genetic variants with sunitinib outcome in metastatic renal cell carcinoma patients

**DOI:** 10.18632/oncotarget.13597

**Published:** 2016-11-25

**Authors:** Xiaoyan Liu, Jesse J. Swen, Epie Boven, Daniel Castellano, Hans Gelderblom, Ron H.J. Mathijssen, Cristina Rodríguez-Antona, Jesus García-Donas, Brian I. Rini, Henk-Jan Guchelaar

**Affiliations:** ^1^ Department of Clinical Pharmacy and Toxicology, Leiden University Medical Center, Leiden, The Netherlands; ^2^ Institute of Clinical Pharmacology, Qilu Hospital of Shandong University, Jinan, China; ^3^ Department of Medical Oncology, VU University Medical Center, Amsterdam, The Netherlands; ^4^ Oncology Department, Hospital Universitario 12 de Octubre, Madrid, Spain; ^5^ Spanish Oncology Genitourinary Group (SOGUG), Madrid, Spain; ^6^ Department of Medical Oncology, Leiden University Medical Center, Leiden, The Netherlands; ^7^ Department of Medical Oncology, Erasmus MC Cancer Institute, Rotterdam, The Netherlands; ^8^ Hereditary Endocrine Cancer Group, Spanish National Cancer Research Centre (CNIO), Madrid, Spain; ^9^ ISCIII Center for Biomedical Research on Rare Diseases (CIBERER), Madrid, Spain; ^10^ Oncology Unit, Clara Campal Comprehensive Cancer Center, Madrid, Spain; ^11^ Department of Solid Tumor Oncology, Cleveland Clinic Taussig Cancer Institute (CCF), Cleveland, Ohio, USA

**Keywords:** sunitinib, metastatic renal cell carcinoma, *VEGFR1*, validation study, meta-analysis

## Abstract

*VEGFR1* rs9582036 and rs9554320 were previously reported the association with sunitinib progression-free survival (PFS) and overall survival (OS) in patients with metastatic renal cell carcinoma (mRCC). Hereafter, the association of both single nucleotide polymorphisms (SNPs) with PFS/OS was confirmed in two independent mRCC cohorts. The aim of the current study was to validate the associations of both SNPs with sunitinib outcome in three independent well-characterized cohorts (SUTOX, CCF and SOGUG) including 286 sunitinib-treated mRCC patients, as well as to perform a meta-analysis of current and published data combined. We found that rs9582036 and rs9554320 showed a significant association with sunitinib PFS in the CCF cohort (HR: 0.254, 95%CI: 0.092-0.703; P=0.008 and HR: 0.430, 95%CI: 0.200-0.927; P=0.031, respectively). Patients with the variant genotype of rs9582036 and rs9554320 had a shorter median PFS. No significant association of both SNPs with sunitinib PFS or OS was detected in either the SUTOX or SOGUG cohort. After the combination of all available data into a meta-analysis, the association of both SNPs with sunitinib PFS or OS did not achieve the threshold for statistical significance. Our findings suggest that, although *VEGFR1* rs9582036 and rs9554320 are involved in sunitinib therapy outcome, its clinical use as biomarkers for prediction of sunitinib outcome in mRCC patients is limited, due to inconsistent findings when analyzing all existing studies together.

## INTRODUCTION

Renal cell carcinoma (RCC) is among the top 10 most common malignancies in men world-wide [1]. There are several subtypes of RCC, but clear cell RCC represents 80-85% of all cases [2]. Metastatic spread has occurred in 25-30% of patients by the time of initial diagnosis [3]. Surgical resection is curative in the majority of RCC patients [4], but patients with advanced or metastatic renal cell carcinoma (mRCC) are candidate for systematic therapy [5].

Studies have shown that vascular endothelial growth factor (VEGF) is highly expressed in RCC, highlighting the fact that RCC is a VEGF-driven disease whose development is directly linked to VEGF overexpression and angiogenesis [6]. In recent years, targeted therapy including the VEGF-inibitor bevacizumab, VEGFR tyrosine kinase inhibitors (TKIs) or mammalian target of rapamycin (mTOR) inhibitors has largely replaced cytokine therapy. Sunitinib, an oral TKI inhibiting VEGFRs 1, 2, and 3, platelet-derived growth factor receptor (PDGFR) α and β, KIT, Fms-like tyrosine kinase 3 receptor (FLT3), and the receptor encoded by the ret proto-oncogene (RET), has been approved by the Food and Drug Administration (FDA) in 2006 and became the first-line treatment for mRCC patients. The median progression-free survival (PFS) has improved considerably from 5 months with interferon-alpha to 11 months with sunitinib [7]. However, only 31% of the patients have complete or partial response and intrinsic resistance is observed in approximately 21% of the patients [7]. The most common adverse events of sunitinib treatment are leukopenia (78%), anemia (71%), thrombocytopenia (60%), diarrhea (53%), fatigue (51%), hypertension (24%) and hand-foot syndrome (20%) [7]. Most adverse events are of grade 1 or 2, and typically manageable with standard medical interventions, but 32% of the patients need dose reduction due to grade 3 or 4 adverse events [8]. Given the large inter-individual variability, the establishment of pharmacogenetic markers to predict the response to sunitinib treatment is a highly desirable goal [8, 9].

In recent years, several single nucleotide polymorphisms (SNPs) in VEGF pathway have been described the association with sunitinib outcome in mRCC patients [8, 10–15]. However, there is a lack of validated biomarker which could predict sunitinib outcome and guide individualized therapy. In 2014, Beuselinck *et al.* explored the association of the *VEGFR1* rs9582036 A>C in 91 mRCC patients treated with sunitinib [16]. Patients with the CC-variant in rs9582036 A>C showed a lower response rate (0% *vs.* 46%) as well as a shorter PFS (10 *vs.* 18 months) and OS (14 *vs.* 31 months) compared to patients with an AC/AA genotype. Besides, Beuselinck *et al.* have reported that patients with the AA-variant in *VEGFR1* rs9554320 C>A had a shorter PFS (12 *vs.* 21 months) and OS (22 *vs.* 34 months) compared to patients with an AC/CC genotype [16]. After correction for covariates, rs9582036 remained significantly associated with OS (HR: 0.25; 95%CI: 0.08-0.80; P=0.008) and rs9554320 with PFS (HR: 0.37; 95%CI: 0.18-0.76; P=0.005) [16]. Hereafter, Dornbusch *et al.* investigated the associations of both SNPs in a cohort of 121 sunitinib-treated mRCC patients but found inconsistent results. It was confirmed that patients with a CC-variant genotype of *VEGFR1* rs9582036 had a shorter OS compared to patients with an AA/AC genotype (16 vs 42 months; HR: 0.24; 95%CI: 0.10-0.60; P= 0.002), but could not replicate the association of rs9582036 with PFS or rs9554320 with PFS and OS [17]. Lately, Beuselinck *et al.* successfully validated the association of both SNPs in another independent validation cohort of 69 mRCC patients treated with sunitinib. After pooling patients from the discovery and validation cohort, both SNPs remained significant association with PFS and OS [18].

Located in the intron region, both SNPs may cause a change of VEGFR1 expression. It has been reported that minor allele of rs9582036 reduces transcriptional activity and decreases VEGFR1 expression [19]. But this correlation has not yet been validated by either other researchers or Beuselinck et al [18]. So far, it is unclear of the function of both SNPs and it is uncertain whether or not the rs9582036 and rs9554320 variants can be used as genetic predictors for the outcome of sunitinib treatment. In this study, we aimed to assess the role of the two *VEGFR1* SNPs rs9582036 and rs9554320 with regard to their association with sunitinib efficacy in three independent well-characterized cohorts of mRCC patients. Moreover, we performed a meta-analysis including all the available data (n=564 mRCC patients) on the possible association of both SNPs with sunitinib efficacy in mRCC patients.

## RESULTS

Patient characteristics of the three cohorts are summarized in Table 1. The median age in the SUTOX, SOGUG and CCF cohort was 60, 64 and 61 years, respectively. More than 66% of included patients were male and the majority (> 94%) of patients were Caucasian. The median follow-up was 50 months for SUTOX, 52 months for CCF and 40 months for SOGUG cohort. The call rates of genotyping were higher than 95% in the three cohorts. There were no significant deviations from Hardy-Weinberg equilibrium (P>0.05). The minor allele frequency (MAF) of rs9582036 in the three cohorts and MAF of rs9554320 in the SUTOX and CCF cohorts were comparable with the data of HapMap-CEU in dbSNP (Table 2). However, the MAF of rs9554320 in the SOGUG cohort was significantly lower than that of HapMap-CEU in dbSNP (P=0.013).

**Table 1:** Patients characteristics from two published reports and three current cohorts

Characteristics	Beuselinck [18]	Dornbosch [17]	SUTOX	CCF	SOGUG
Number of patients	157	121	124	74	88
Median age at sunitinib start (years)	59^a^	59^b^	60	61	64
Male	113 (72%)	95 (79%)	82 (66%)	51 (69%)	61 (69%)
Caucasian	NA	121 (100%)	116 (94%)	73 (99%)	86 (98%)
Prior treatment	NA	5 (4%)	28 (23%)	22 (30%)	0
Cytokines	NA	0	25	15	0
Sorafenib	NA	5	1	5	0
Other therapies	NA	0	2	2	0
Metastasis					
Lung	117 (75%)	NA	82 (66%)	44 (60%)	61 (69%)
Liver	29 (18%)	NA	31 (25%)	6 (8%)	13 (15%)
Bone	57 (36%)	NA	39 (32%)	19 (26%)	23 (26%)
Brain	11 (7%)	NA	3 (2%)	9 (12%)	2 (2%)
Mean number of metastases	2.31	NA	2.24	1.80	1.98
Risk group*					
Favourable	30 (19%)	NA	25 (20%)	28 (38%)	10 (11%)
Intermediate	95 (64%)	NA	66 (53%)	39 (53%)	57 (65%)
Poor	29 (20%)	NA	33 (27%)	7 (10%)	21 (24%)
Median follow-up (month)	77	25	50	52	40
Median TTP/PFS (month)	12	14	16	33	17
Median OS (month)	27	25	23	53	27

a Mean age at initial diagnosis;

b Median age at surgery;

* Risk group by Beuselinck *et al*. was categorized by the International Metastatic Renal-Cell Carcinoma Database Consortium (IMDC) criteria, while SUTOX, CCF and SOGUG cohorts were categorized in Heng prognostic risk groups [25]. Both criteria include the same variables including poor WHO performance status (≥2), low haemoglobin (< lower limit of normal), high calcium (> upper limit of normal) and time from initial diagnosis to treatment with sunitinib (< 1 year), neutrophil count (> upper limit of normal) and thrombocytes (> upper limit of normal).

NA: not available; TTP: time to progression; PFS: progression-free survival; OS: overall survival; SUTOX: five medical centres in The Netherlands; SOGUG: Spanish Oncology Genitourinary Group medical centres including15 Spanish participating hospitals; CCF: Cleveland Clinic Foundation Taussig Cancer Institute.

**Table 2:** The minor allele frequency of ***VEGFR1*** rs9582036 and rs9554320

SNPs	Genotype	SUTOX (n=124)	CCF (n=74)	SOGUG (n=88)	Beuselinck [18] (n=157)	Dornbusch [17] (n=121)	HapMap-CEU (n=226)
rs9582036	AA	68	33	44	78	67	118
	AC	49	36	32	64	43	84
	CC	6	5	7	13	10	24
	HWE (*P* value)	0.45	0.24	0.73	0.98	0.48	0.13
	MAF	0.248	0.311	0.277	0.292	0.260	0.292
rs9554320	CC	46	23	39	57	41	62
	CA	61	40	36	68	57	122
	AA	15	10	12	27	23	42
	HWE (*P* value)	0.45	0.26	0.43	0.39	0.713	0.19
	MAF	0.373	0.411	0.345	0.404	0.426	0.456

SUTOX: five medical centres in The Netherlands; SOGUG: Spanish Oncology Genitourinary Group medical centres including 15 Spanish participating hospitals; CCF: Cleveland Clinic Foundation Taussig Cancer Institute; HapMap-CEU represents Utah residents with Northern and Western European ancestry from the CEPH (Centre d'Etude du Polymorphisme Humain) collection, which was collected in 1980; HWE: Hardy-Weinberg equilibrium; MAF: minor allele frequency.

### Replication cohort

We analysed the association of both SNPs with sunitinib PFS and OS in SUTOX, CCF and SOGUG cohorts. *VEGFR1* rs9582036 and rs9554320 showed similar associations with PFS in the CCF cohort as in previous studies. In the Kaplan-Meier analysis, patients with the variant CC genotype of rs9582036 from the CCF cohort had a dramatically shorter median PFS compared to patients with an AA/AC genotype (16.8 months *versus* 35.9 months, P=0.047). In the multivariate Cox-regression analysis corrected for age, gender and Heng prognostic risk group, rs9582036 and rs9554320 remained significantly associated with PFS in the CCF cohort (HR: 0.254, 95%CI: 0.092-0.703; P=0.008 and HR: 0.430, 95% CI: 0.200-0.927; P=0.031, respectively). No significant difference in PFS was observed in the SUTOX and SOGUG cohorts for *VEGFR1* rs9582036 or rs9554320. No significant association of both SNPs with OS was found in the three cohorts (Table 3).

**Table 3:** Association analyses of ***VEGFR1*** rs9582036 and rs9554320 with sunitinib PFS/OS in mRCC patients

	Group	Genotype	N	PFS (month)	P^1^	P^2^	HR	95% CI	OS (month)	P^1^	P^2^	HR	95%CI
rs9582036	SUTOX (n=123)	AA/AC	117	15.5	0.459	0.818	1.154	0.340-3.914	22.6	0.394	0.824	1.147	0.343-3.830
		CC	6	11.1					18.4				
	CCF (n=74)	AA/AC	69	35.9	**0.047**	**0.008**	**0.254**	**0.092-0.703**	52.5	0.869	0.321	0.528	0.150-1.862
		CC	5	16.8					67.5				
	SOGUG (n=83)	AA/AC	76	11.7	0.501	0.535	1.369	0.507-3.700	25.6	0.524	0.533	1.472	0.437-4.954
		CC	7	14.6					19.5				
rs9554320	SUTOX (n=122)	CC/AC	107	15.5	0.890	0.716	0.878	0.435-1.771	22.1	0.459	0.695	1.164	0.544-2.492
		AA	15	16.0					22.6				
	CCF (n=73)	CC/AC	63	36.6	**0.189**	**0.031**	**0.430**	**0.200-0.927**	54.3	0.974	0.275	0.586	0.224-1.531
		AA	10	21.4					52.5				
	SOGUG (n=87)	CC/AC	75	11.7	0.519	0.757	1.126	0.532-2.382	26.7	0.260	0.338	1.599	0.612-4.174
		AA	12	14.6					NR				

SUTOX: five medical centres in The Netherlands; SOGUG: Spanish Oncology Genitourinary Group medical centres including 15 Spanish participating hospitals; CCF: Cleveland Clinic Foundation Taussig Cancer Institute; P^1^ from log-rank test; P^2^ from multiple cox regression analysis corrected by age, gender and Heng prognostic risk group [25]; NR: not reach; PFS: progression-free survival; OS: overall survival; HR: hazard ratio; CI: confidence interval

### Meta-analysis

**Figure 1:**
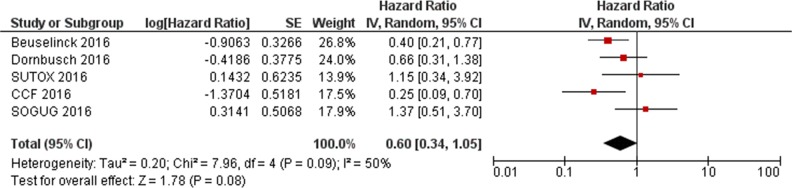
Forest plot for association of *VEGFR1* rs9582036 with sunitinib progression-free survival

**Figure 2:**
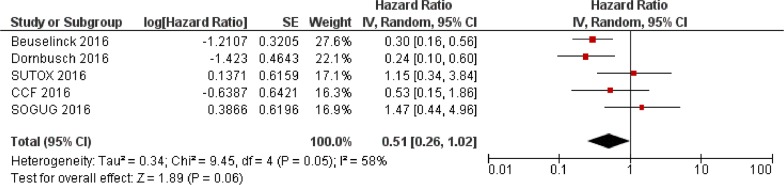
Forest plot for association of *VEGFR1* rs9582036 with sunitinib overall survival

**Figure 3:**
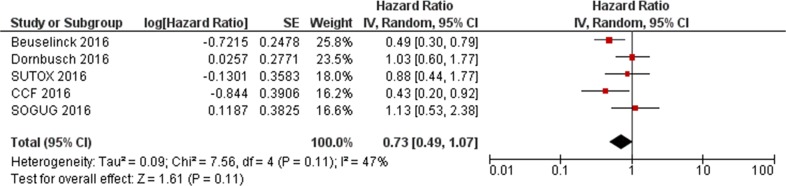
Forest plot for association of *VEGFR1* rs9554320 with sunitinib progression-free survival

**Figure 4:**
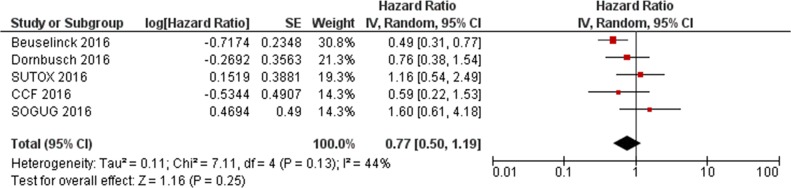
Forest plot for association of *VEGFR1* rs9554320 with sunitinib overall survival

Data from the present study and the two previous reports [17, 18] were extracted and pooled to evaluate the effect of *VEGFR1* rs9582036 and rs9554320 on sunitinib PFS and OS. In the total population (n=564), the association of rs9582036 with PFS (HR: 0.60; 95%CI: 0.34-1.05; P=0.08; Figure 1) or OS (HR: 0.51; 95%CI: 0.26-1.02; P=0.06; Figure 2) did not reach the threshold for statistical significance, nor did the association of rs9554320 with sunitinib PFS (HR: 0.73; 95%CI: 0.49-1.07; P=0.11; Figure 3) and OS (HR: 0.77; 95%CI: 0.50-1.19; P=0.25; Figure 4).

## DISCUSSION

Exploratory studies have suggested that *VEGFR1* rs9582036 and rs9554320 may be associated with PFS and OS in sunitinib-treated mRCC patients [16–18], but results were inconclusive. Whether these SNPs are useful as a predictive biomarker for sunitinib efficacy in mRCC patients remains an open question. The current study aimed to provide a conclusive answer to this question by replication of the analysis in three independent well-characterized cohorts (SUTOX, CCF and SOGUG) with a total of 286 mRCC patients and performing a meta-analysis of all available data (n=564 mRCC patients).

While associations of both SNPs with PFS could be confirmed in the CCF cohort (n=74), no correlation of both SNPs with PFS/OS was found in either the SUTOX or SOGUG cohort. In the meta-analysis of all available data (n=564), the association of the *VEGFR1* polymorphisms with PFS or OS did not reach the threshold for statistical significance. Therefore, it can be concluded that *VEGFR1* rs9582036 and rs9554320 have a small effect on the prediction of sunitinib outcome in mRCC patients and thus have only limited use as genetic predictors of sunitinib efficacy in this disease.

In recent years, genotyping technology has developed rapidly and as a result the number of SNPs tested as potential markers for therapy selection has increased massively. However, it has been shown repeatedly that the findings in the discovery pharmacogenetic studies are difficult to replicate [20–23]. A major reason of replication failure is the large heterogeneity among studies. Of note, the data from the present and previous studies were collected retrospectively from various centres across the world and patients were not enrolled in a designated pharmacogenetic study. Use of observational data may have the disadvantage of less systematic collection of clinical data and a high heterogeneity among patients as compared to a clinical trial setting. However, the cohorts in the current study were all well characterized. Further, the observational data may be better representative for the real world clinical practice.

Due to daily clinical practice, inclusion and exclusion criteria were not as strict as in a clinical trial setting, resulting in different proportions of patients with prior treatment among the five cohorts. It has been mentioned by Beuselinck *et al.* that 23% of patients received immunotherapy prior to sunitinib [16, 18]. Dornbusch *et al.* have included 4% of patients treated with sorafenib before sunitinib [17]. In the present study, 28 (23%) and 22 (30%) patients from SUTOX and CCF cohorts received prior treatments, including immunotherapy (n=25 and 15, respectively), sorafenib (n=1and 5, respectively) or other (n=2 and 2, respectively). All patients from the SOGUG cohort were treated with sunitinib first-line. Obviously, prior treatments can influence the efficacy of subsequent therapy. However, as second-line therapy after cytokine failure, sunitinib demonstrated comparable antitumor activity to sunitinib given as first-line [7], which makes it rational to include cytokine-refractory patients in the current study. The number of patients in the present study having received pre-treatment with sorafenib was small. In a *post hoc* analysis, we excluded all pre-treated patients and similar results were found (data not shown).

In previous studies, different covariates were used for multivariate analysis in each study. For example, Beuselinck *et al.* have considered International Metastatic Renal-Cell Carcinoma Database Consortium (IMDC) prognostic score as covariates [18], while Dornbusch *et al.* have corrected multivariate analyses by tumor, node and metastasis (TNM) stage and tumor grade [17]. We reanalysed our data by using the same covariates as the previous studies, but did not see any significant associations (data not shown).

DNA was mostly isolated from kidney tissues in both previous studies (71% in Beuselinck cohort and 67% in Dornbosch cohort) [17, 18], whereas our DNA samples were all derived from whole blood, serum or plasma [10, 24]. Even though the sources of DNA were partly different and the MAF of rs9554320 of the SOGUG cohort was lower than that of HapMap-CEU, there was no significant difference in the MAF of both SNPs across five cohorts. Therefore, we believe that differences in source of DNA in our studies as compared to the previous studies do not explain the failure of replication.

Besides the above-mentioned factors, we also compared the treatment regimen, follow-up period, period of clinical practice taken (ranging from 2004-2015), and patients characteristics, for example, distribution of patients risk groups among the five cohorts, but did not observe major differences. Further, we do not have the access to the published data in detail and lack sufficient information on the subsequent treatment. The origin of inconsistencies in findings remains unknown. Due to the fact that the associations of both SNPs with sunitinib outcome were observed in small cohorts, the positive effects may be present by chance.

In conclusion, the association of *VEGFR1* rs9582036 and rs9554320 with the outcome of sunitinib in mRCC patients did not reach the threshold for statistical significance, and therefore, both genetic variants have limited use as biomarkers for prediction of sunitinib efficacy.

## MATERIALS AND METHODS

### Study population

Three cohorts of clear cell mRCC patients treated with sunitinib between the years 2004 and 2012 were included in this study. Patient characteristics have been described previously [10, 24]. The SUTOX cohort consisted of 124 mRCC patients from five medical centres in The Netherlands, the SOGUG cohort contained 88 mRCC patients from Spanish Oncology Genitourinary Group (SOGUG) medical centres (15 Spanish participating hospitals) and the CCF cohort contained 74 mRCC patients from the Cleveland Clinic Foundation (CCF) Taussig Cancer Institute. All mRCC patients received 50 mg, 37.5 mg or 25 mg sunitinib for at least one cycle in a 4-week on/2-week off schedule or a continuous dosing regimen. The study was conducted in accordance with the Declaration of Helsinki and approved by the medical ethics review board of all participating cohorts. Patients from the SOGUG and CCF cohorts provided their written informed consent for participation. SUTOX samples were anonymised by a third party according to the instructions stated in the Codes for Proper Use and Proper Conduct in the Self-Regulatory Codes of Conduct (www.federa.org) [10, 24].

### Study endpoints

The primary endpoint PFS, was defined as the time in months between the first day of sunitinib treatment and the date of progressive disease (PD) or time of last follow-up according to Response Evaluation Criteria in Solid Tumours (RECIST, v.1.0 or v1.1). Secondary endpoint was OS, which was measured from the first day of sunitinib treatment until death or time of last follow-up [10, 24].

### Genotyping

Genomic DNA was extracted from whole blood, serum, plasma or peripheral blood mononuclear cell samples [10, 24]. Two *VEGFR1* SNPs rs9582036 A>C and rs9554320 C>A were genotyped using Taqman probes (Applied Biosystems, Nieuwerkerk aan den IJssel, the Netherlands) on the LightCycler 480 (LC480) Real-Time PCR Instrument (Roche Applied Science, Almere, The Netherlands) in SUTOX and CCF samples, and using KASPar SNP genotyping system (Kbiosciences, Hoddesdon, UK) and the sequence Detection System 7900HT (Applied Biosystems, Foster City, CA, USA) in SOGUG samples. Cross validation between assays was described in our previous studies and confirmed analytical validity [10, 24].

### Statistical analysis

Hardy-Weinberg equilibrium test was performed for both SNPs in each cohort. Survival analyses of PFS and OS were assessed by Kaplan-Meier method using log-rank test. The associations of both SNPs with sunitinib PFS and OS were tested in each replication cohort using multivariate Cox-regression model. Hazard ratio (HR) and 95%CI for each SNP in each cohort was estimated by SPSS Statistical Package for Windows (version 23 Armonk, NY: IBM Corp). Because the present study is a replication study, the recessive genetic model was assumed in order to keep consistency with previously published studies. Well-established covariates age, gender and Heng prognostic risk group [25] were included as covariates in the multivariate model for PFS and OS.

Due to the lack of HR from multivariate analysis for validation cohort (n=69) from Beuselinck *et al*, only pooled cohort (n=157) was included in the meta-analysis. The estimated HR and 95%CI from the present study and previous reports [17, 18] were pooled into a meta-analysis using the Review Manager software (RevMan, version 5.3). Heterogeneity of effects among the individual cohorts was assessed using the I^2^ index of heterogeneity and by Cochran's Q statistic. The random effect model was used for meta-analysis in all cases, because when studies were gathered from the published literature, the random effects model is generally a more plausible match [26]. The Z test was used to determine the significance of the pooled HR. Missing data were kept missing except those used for evaluation of Heng prognostic risk group, which were replaced by single imputation. To test this action, the multivariate analyses were performed with and without the replacement of the patients with missing factors in the Heng prognostic risk group. Similar results were generated, indicating that the replacement was legitimate.
